# EGFR Gene Variants Are Associated with Specific Somatic Aberrations in Glioma

**DOI:** 10.1371/journal.pone.0047929

**Published:** 2012-12-07

**Authors:** Carl Wibom, Soma Ghasimi, Peter Van Loo, Thomas Brännström, Johan Trygg, Ching Lau, Roger Henriksson, Tommy Bergenheim, Ulrika Andersson, Patrik Rydén, Beatrice Melin

**Affiliations:** 1 Department of Radiation Sciences, Oncology, Computational Life Science Cluster (CLiC), Umeå University, Umeå, Sweden; 2 Department of Radiation Sciences, Oncology, Umeå University, Umeå, Sweden; 3 Cancer Genome Project, Wellcome Trust Sanger Institute, Hinxton Cambridge, United Kingdom; 4 Department of Medical Biosciences, Pathology, Umeå University, Umeå, Sweden; 5 Department of Chemistry, Computational Life Science Cluster (CLiC), Umeå University, Umeå, Sweden; 6 Department of Pediatrics and Hematology, Baylor College of Medicine, Houston, Texas, United States of America; 7 Department of Oncology, Karolinska Institute, Stockholm, Umeå, Sweden; 8 Pharmacology and Clinical Neuroscience, Umeå University, Umeå, Sweden; 9 Computational Life Science Cluster (CLiC), Department of Mathematics and Mathematical Statistics, Umeå University, Umeå, Sweden; 10 Department of Human Genetics, VIB and KU Leuven, Leuven, Belgium; University of California-San Francisco, United States of America

## Abstract

A number of gene variants have been associated with an increased risk of developing glioma. We hypothesized that the reported risk variants may be associated with tumor genomic instability. To explore potential correlations between germline risk variants and somatic genetic events, we analyzed matched tumor and blood samples from 95 glioma patients by means of SNP genotyping. The generated genotype data was used to calculate genome-wide allele-specific copy number profiles of the tumor samples. We compared the copy number profiles across samples and found two EGFR gene variants (rs17172430 and rs11979158) that were associated with homozygous deletion at the CDKN2A/B locus. One of the EGFR variants (rs17172430) was also associated with loss of heterozygosity at the EGFR locus. Our findings were confirmed in a separate dataset consisting of matched blood and tumor samples from 300 glioblastoma patients, compiled from publically available TCGA data. These results imply there is a functional effect of germline EGFR variants on tumor progression.

## Introduction

Genome-wide association studies (GWAS) have identified common genetic variants that are likely to be involved in the etiology of glioma. There are three published GWAS to date that have identified eight different loci associated with glioma risk [1], [2], [3], including variants annotating key genes in glioma progression, such as the epidermal growth factor receptor (EGFR), and the tumor suppressor gene CDKN2A (alias p14, p16, and ARF). In addition to the GWAS, two separate candidate gene studies have been performed [4], [5], resulting in a number of putative risk variants associated with glioma susceptibility.

The Cancer Genome Atlas (TCGA) has published a comprehensive genomic analysis of 206 glioblastoma cases [6]. This work highlights three pathways, including 20 genes, of particular interest in glioma tumorigenesis. Four out of eight of the risk variants reported in the GWAS studies map to genes listed by the TCGA report. Each locus that the GWAS risk variants map to, and their involvement in glioblastoma tumorigenesis, is summarized in a review by Melin [7].

Many of the loci harboring the risk variants (Table 1) can be directly or indirectly linked to genomic stability. First, most obvious are the two genes involved in regulation of telomeres (RTEL1 and TERT). RTEL1 is directly involved in maintenance of genome stability, through suppression of homologous recombination [8], and TERT expression is shown to correlate with enhanced genome stability and DNA repair [9]. Second, the CDKN2A/CDKN2B gene products are involved in RB-signaling, and as such they are ultimately involved in regulation of genomic stability through cell cycle control. Third, EGFR acts as an early activator of transcription in the RAS signaling pathway, where dysfunctional RAS regulation is implicated in destabilization of the karyotype, especially in the absence of p53 [10]. Lastly, ERBB2 is included in the same growth factor receptor family as EGFR and interacts physically with EGFR by dimerization [11]. The functions of PHDLB1 and CCDC26 are less well characterized. Variations within these genes are associated especially with low grade glioma [12], [13].

**Table 1:** Risk gene variants.

							Discovery (UMU)							Validation (TCGA)						
Risk variant	Chr	Position	Gene	Major allele	Risk allele	Ref.	Surrogate	Position	LD (r∧2)	Major allele	Risk allele	n (major)	n (rare+hz)	Surrogate	Position	LD (r∧2)	Major allele	Risk allele	n (major)	n (rare+hz)
rs2736100	5	1339516	TERT	A	C	[2]						24	57						46	239
rs2252586	7	54946418	EGFR	G	A	[1]	rs6945082	54925952	0.883	G	A	38	43						116	169
rs6969537	7	55049912	EGFR	G	G	[5]						55	26						222	63
rs17172430	7	55090144	EGFR	G	G	[4]	rs1015793	55081810	0.742	A	A	60	21						236	49
rs11979158	7	55126843	EGFR	A	A	[1]	rs10245472	55114972	1.000	G	G	57	24						209	76
rs4947979	7	55163119	EGFR	A	A	[4]						44	37						184	101
rs4295627	8	130754639	CCDC26	A	C	[2]	rs6470745	130711103	1.000	A	G	43	38						198	87
rs1412829	9	22033926	CDKN2B	A	G	[3]	rs634537	22022152	1.000	A	C	17	64						89	196
rs4977756	9	22058652	CDKN2A-CDKN2B	A	G	[2]						18	63						92	193
rs498872	11	117982577	PHLDB1	G	A	[2]						31	50						131	154
rs1476278	17	35089769	ERBB2	A	G	[4]	rs903502	35083130	1.000	A	G	33	48	rs12150298	35088067	1.000	G	A	122	163
rs2952155	17	35115244	ERBB2	G	A	[4]	rs9635726	35273667	0.678	G	A	44	37	rs9635726	35273667	0.678	G	A	186	99
rs6010620	20	61780283	RTEL1	G	G	[2], [3]						52	29						201	84

*LD(r∧2)* HapMap linkage disequilibrium (r2) data between used surrogate marker and original risk variant, *n* number, *major* samples homozygous for the major allele, *rare+hz* samples homozygous for the rare allele plus heterozygous samples.

We hypothesized that reported risk variants are associated with genomic instability. To test this hypothesis, we analyzed matched blood and tumor samples from 95 glioma patients by means of SNP genotyping. Based on the SNP genotyping data, we calculated genome-wide allele-specific copy number in the tumor samples. This enabled us to explore possible correlations between germline risk genotypes and frequencies of somatic aberrations.

## Materials and Methods

### Patients and Ethics Statement

This study was based on samples collected from glioma patients diagnosed at Umeå University Hospital, between 1995 and 2008. A total of 197 patients were diagnosed during this period. Ninety-five (95) patients from whom matched blood and tumor samples were available were included in the study. Diagnoses were confirmed by pathology review. This sample set is referred to as the UMU set, and its characteristics are listed in Table 2.

**Table 2:** UMU sample set characteristics.

	All samples	Solved by ASCAT
Total number of patients	95	81
Gender (male/female)	59/36	52/29
Age at dianosis median (yrs)	56 (15–80)	55 (15–80)
Male	57	57
Female	54	53
Histological subtype distribution		
GBM	63	55
Non GBM	32	26

Collection of blood samples, brain tumor tissues and clinico-pathological information from patients was undertaken with written informed consent and the study was approved by our ethical board, in accordance with the Umeå University Hospital guidelines.

### DNA extraction and Genotyping

DNA was extracted from EDTA-venous blood samples using FlexiGene DNA Kit (QIAGEN GmbH, Hilden, Germany) and brain tumor tissues using QIAmp DNA Mini Kit (QIAGEN GmbH, Hilden, Germany) methodologies. Genotyping was conducted by the SNP&SEQ Technology Platform, Uppsala, Sweden (www.genotyping.se) using Illumina HumanOmni1-Quad BeadChips according to the manufacturer's protocols.

### TCGA data

The validation dataset for this study was compiled from publically available TCGA data. Illumina idat-files from matched tumor and blood samples were downloaded (13 December 2011) for 334 GBM patients analyzed on the Illumina HumanHap550 array. Samples from 32 patients (all from the same sample plate) were excluded due to a large proportion of failed probes (>5%). Furthermore, we excluded two additional patients due to probable sample mix-ups (the blood raw data profiles appeared very similar to typical tumor samples). In total we found matched tumor and blood samples from 300 GBM patients eligible to use as a validation set.

### Data Pretreatment

Generated intensity data was imported into GenomeStudio software. The GenCall Score cutoff was set to 0.15. Log R ratio (LRR) and B allele frequency (BAF) data from each sample and probe was subsequently exported. To avoid downstream difficulties with segmentation, we removed LRR and BAF data from W-probes with LRR<−2 and replaced them with missing value. This was done individually for each sample in the UMU-dataset. The HumanHap550 array does not contain W-probes, hence this does not apply to the TCGA data. Lastly, we adjusted for GC-waves in both datasets [14].

### Allele-Specific Copy Number

**Figure 1:**
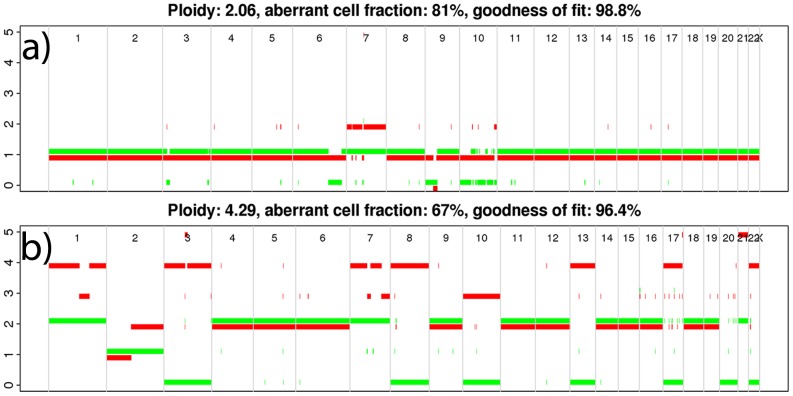
ASCAT-profiles. Whole genome ASCAT-profiles from two samples in the UMU dataset; one diploid (**a**) and one tetraploid (**b**). Green represents the allele with the lower copy number, and red represents the allele with the higher copy number (the colors are slightly offsetted to avoid overlap, red downwards and green upwards).

We used the ASCAT-algorithm [15] (version 2.0) to calculate genome-wide allele-specific copy number individually for each sample (Fig. 1). ASCAT also estimates tumor cell content and tumor cell ploidy.

### Copy number analyses

**Figure 2:**
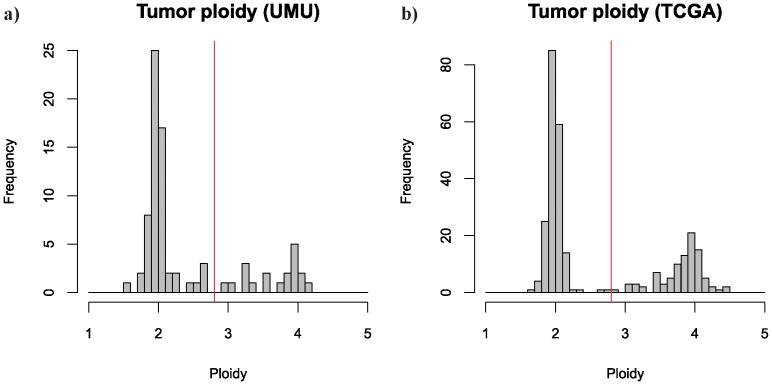
Tumor ploidy. ASCAT calculated tumor ploidy for 81 UMU samples (**a**) and 285 TCGA samples (**b**). Samples with ploidy >2.8 were classified as tetraploid-like and samples with ploidy < = 2.8 as diploid-like.

Each individual probe was assigned to at least one of eight types of genomic events (Table 3). In order to account for aneuploidy due to whole-genome duplication by endoreduplication and make copy number comparisons over samples with different ploidy more biologically relevant, we first assigned samples either a diploid-like or tetraploid-like subclass, and divided the copy numbers by two for tetraploid-like samples. To classify samples as tetraploid- or diploid-like, we used the ASCAT sample ploidy estimation and set the cutoff to 2.8, as samples with a sample ploidy above this threshold seem to have undergone whole-genome duplication, contrary to samples with lower sample ploidy (Fig. 2).

**Table 3:** Description of genomic events.

Genomic events	Definition^a^
Normal	nA = 1 AND nB = 1
Loss	nTot <2
Increased copy number	nTot >2
Loss of heterozygosity (LOH)	nA = 0 OR nB = 0
Copy number neutral events (CNNE)	nA≠1 AND nB≠1 AND nTot = 2
Homozygous deletion (HD)	nTot = 0
Amplification	nTot > = 8
Simultaneous LOH and increased copy number	(nA = 0 OR nB = 0) AND nTot >2

a nTot = nA + nB, where nA and nB represent calculated copy numbers for the separate alleles at a given probe.

In the discovery phase, performed on UMU-data, we used two complementary approaches to study correlation between risk variants and the specified genomic events; one global and one focused on selected genes of interest. The global approach was designed to explore correlations anywhere in the genome, with the prerequisite that the event frequency was relatively large. The genes-of-interest approach was designed to explore a set of predefined genes, regardless of event frequency. All steps of the analyses were performed in R (www.R-project.org).

### Global approach

For each individual probe, we calculated the frequency of a given event in all samples. We then calculated an event frequency cutoff, to locate recurring events in our sample series. The event frequency cutoff was calculated based on permutations of genomic positions. After the genomic positions had been permuted, individually for each sample, the event frequency at each probe was determined. Based on the generated random frequency data, an intermediate event frequency cutoff was set using a false positive rate of 0.5% (which we estimate correspond to a true discovery rate of 90 to 99%). The permutation of genomic positions was repeated a total of 100 times, and we used the mean value of the intermediate event frequency cutoffs as our final cutoff to call recurrent events. X-chromosome data was left out of the permutation procedure.

We split the dataset into two groups based on a given risk variant (Table 1); samples homozygous for the major allele constituted one group and samples homozygous for the rare allele plus heterozygous samples constituted the other group. In case the risk variant we aimed to investigate was not present on the SNP array, we used a surrogate marker that was in linkage disequilibrium with the original risk variant (Table 1). The surrogate marker was selected from available SNPs as the one with the largest r2-value, based on HapMap data. We then tested the hypothesis that the group containing the risk allele displayed a higher event frequency than the other group, by applying one sided Fisher's Exact tests to the frequency data of each probe. This was performed only on probes within regions where the risk group displayed event frequencies above the event frequency cutoff. Genomic regions with p-value<cutoff (defined below) in the UMU dataset were shortlisted for validation in the TCGA dataset.

The p-value cutoff was determined through permutation. In each permutation, we randomly assigned all samples to one of the two groups (while maintaining constant group sizes), and thereafter calculated p-values across the genome, as described above. The permutation was repeated 100 times, and for each run the lowest recorded p-value was stored. The p-value cutoff was set to the 95^th^ percentile of the stored p-values from the permutation procedure. This procedure is similar to that described in Lystig et al [16].

This approach was repeated for each event of interest, as well as for all risk variants.

### Genes-of-interest approach

The genes of interest (Table 4) were selected from the pathways identified in the TCGA report [6], and supplemented with a few genes of documented biological relevance to glioma tumorigenesis.

**Table 4:** Genes of interest.

Gene	Locus
CDKN2C	chr1:51206954-51212894
MDM4	chr1:202752129-202793871
AKT3	chr1:241729643-242073207
IDH1	chr2:208809197-208828051
PIK3CA	chr3:180349004-180435191
PDGFRA	chr4:54790020-54859169
TERT	chr5:1306286-1348162
PIK3R1	chr5:67547339-67633405
PARK2	chr6:161688579-163068824
EGFR	chr7:55054218-55242525
CDK6	chr7:92072170-92301167
MET	chr7:116099694-116225676
MYC	chr8:128817496-128822862
CCDC26	chr8:130433119-130761667
CDKN2A	chr9:21957750-21965132
CDKN2B	chr9:21992901-21999312
PTEN	chr10:89613174-89718512
PHLDB1	chr11:117982422-118033958
CCND2	chr12:4253162-4284783
CDK4	chr12:56427776-56432497
MDM2	chr12:67488237-67525479
RB1	chr13:47775883-47954027
IDH2	chr15:88428215-88446712
TP53	chr17:7512444-7531593
NF1	chr17:26446070-26728821
ERBB2	chr17:35109779-35138441
RTEL1	chr20:61759606-61798050

For each gene of interest, each sample was classified as positive or negative for a given event. Samples were classified as positive if >50% of the probes within the locus of the gene were positive, and vice versa. We divided the samples into two groups, based on the same principle used in the global approach. Subsequently we applied a one sided Fisher's Exact test to the contingency table of samples with and without event, to test the hypothesis that the group containing the risk allele displayed a higher event frequency than the other group. Gene/event combinations with p<0.05 in the UMU dataset were shortlisted for validation in the TCGA data set.

### Validation

All events found in the discovery phase were subsequently validated in the TCGA dataset. We used the same method as for the genes-of-interest approach. I.e., for each combination of event and region/gene of interest that was selected for validation, we classified all TCGA samples as either positive or negative for the given event within the region/gene of interest. To be classified as positive, >50% of all probes within the region/gene of interest had to be positive. We thereafter split the dataset in two and performed a one-sided Fisher's Exact test, as previously described.

## Results

We inferred copy number profiles of glioma tumor cells from SNP array data, accounting for non-aberrant cell admixture and tumor aneuploidy, using the ASCAT algorithm [15]. We obtained ASCAT profiles (i.e. whole-genome allele-specific copy number profiles) (Fig. 1) for 81 of 95 samples (85%) in the UMU discovery dataset, and for 285 of 300 samples (95%) in the TCGA validation dataset. Distributions of sample ploidy were similar for both datasets and indicated that 23% and 33% of the samples had undergone whole-genome duplication in the UMU and TCGA datasets respectively (Fig. 2).

**Figure 3:**
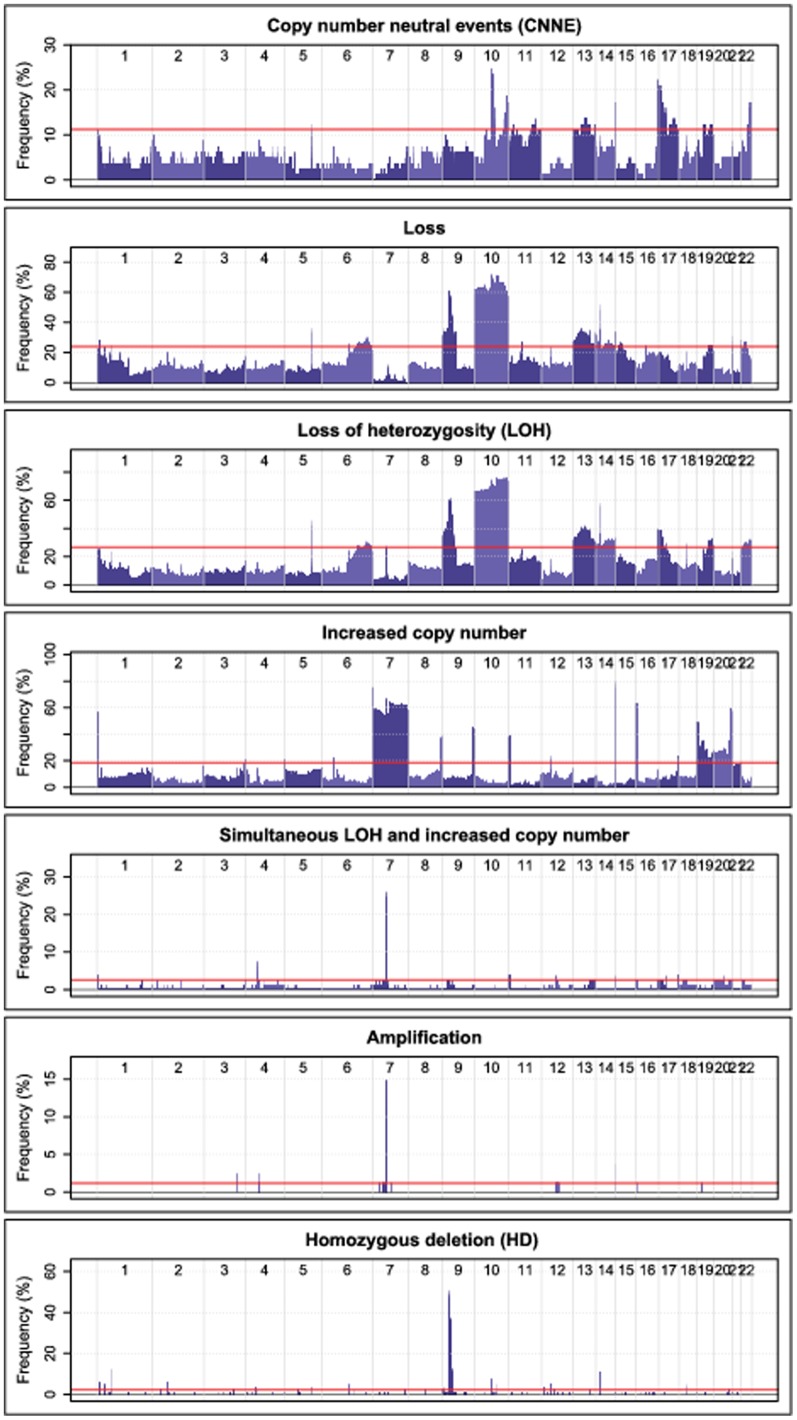
Frequency of genomic events among UMU samples. Frequency (%) is represented on the y-axis and genomic position on the x-axis. The permutation derived event frequency cutoff (used to establish regions with significantly recurring events among the UMU samples) is illustrated by the red line.

**Figure 4:**
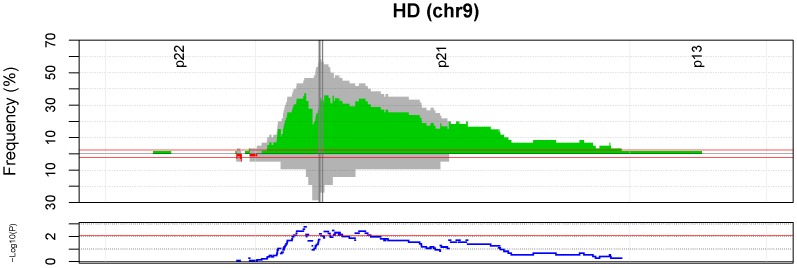
Frequency of homozygous deletion at chr9.p21, among UMU samples. The samples are split into two groups, based on their genotype at the EGFR variant rs1015793. HD frequency among samples homozygous for the major allele is plotted on the upper half of the upper panel (grey bars), and the HD frequency among samples homozygous for the rare allele plus heterozygous samples is plotted on the lower half of the upper panel (grey bars). The frequency difference between the groups is illustrated with overlaying red and green bars. The event frequency cutoff is illustrated by the horizontal red lines. The dark grey, vertical lines represent CDKN2A and CDKN2B. The lower panel illustrates the p-values (−log10 transformed) at each genomic site, with the horizontal red line illustrating the permutation derived p-value cutoff (0.0085).

We calculated the frequency of the defined somatic events over the whole genome in the UMU dataset, and determined regions where each event was significantly recurring (Fig. 3). Within these regions, we tested the hypothesis that samples that carry the germline risk genotype for a given risk variant also display a greater frequency of somatic aberrations. In the discovery phase, we found 59 region/event combinations that were significantly more frequent in the risk group (Table S1). In many cases the same type of event, correlated to the same risk genotype, occurred on genomic regions adjacent to each other, and were only separated due to frequency drops caused by genomic breakpoints in just a few patients. Nine of these events could not be tested in the validation phase, due to a lack of probe coverage in the given region (number of probes < = 2 on the HumanHap550 array). Two of the remaining events were found to be significant also in the validation set. They were both homozygous deletion events within the 9p21.3 region, correlated to the EGFR variant rs17172430 (Fig. 4; Table 5; Table S1).

**Table 5:** Significant correlations between germline gene variants and somatic aberrations.

					UMU						TCGA						
Risk variant	Region	Cytoband	Gene	Event	Variant	n (major)^a^	n (major) event^a^	n(rare+hz)^a^	n(rare+hz) event^a^	P	Variant	n (major)	n (major) event	n(rare+hz)	n(rare+hz) event	P	Approach
rs17172430	chr7:55054218-55242525	7p11.2	EGFR	LOH	rs1015793	60 (41/19)	19 (15/4)	21 (14/7)	2 (2/0)	0.0385	rs17172430	236	57	49	6	0.0455	GOI
rs11979158	chr9:21957750-21965132	9p21.3	CDKN2A	HD	rs10245472	57 (39/18)	33 (26/7)	24 (16/8)	8 (7/1)	0.0374	rs11979158	209	51	76	10	0.0267	GOI
rs11979158	chr9:21992901-21999312	9p21.3	CDKN2B	HD	rs10245472	57 (39/18)	32 (25/7)	24 (16/8)	7 (6/1)	0.0233	rs11979158	209	122	76	32	0.0107	GOI
rs17172430	chr9:21992901-21999312	9p21.3	CDKN2B	HD	rs1015793	60 (41/19)	34 (27/7)	21 (14/7)	5 (4/1)	0.0088	rs17172430	236	134	49	20	0.0300	GOI
rs17172430	chr9:21961989-21978896	9p21.3	MTAP, CDKN2A	HD	rs1015793	60 (41/19)	35 (28/7)	21 (14/7)	5 (4/1)	0.0062	rs17172430	236	135	49	20	0.0264	global
rs17172430	chr9:22010493-22055620	9p21.3	MTAP, CDKN2BAS	HD	rs1015793	60 (41/19)	33 (27/6)	21 (14/7)	4 (3/1)	0.0040	rs17172430	236	132	49	19	0.0210	global

*n* number, *major* samples homozygous for the major allele, *rare+hz* samples homozygous for the rare allele plus heterozygous samples, *event* samples positive for given event, *GOI* genes of interest.

a total number of samples (glioblastoma samples/non-glioblastoma samples).

By the complementary genes-of-interest approach, we found 35 events that were significantly more frequent in the risk group in the discovery phase. Four of these proved significant also in the validation phase (Table S2). One was LOH in the EGFR gene, associated with a risk variant in the EGFR gene (rs17172430). Three were homozygous deletion events in the CDKN2A/B genes, associated with two different risk variants in the EGFR gene (rs17172430 and rs11979158).

**Figure 5:**
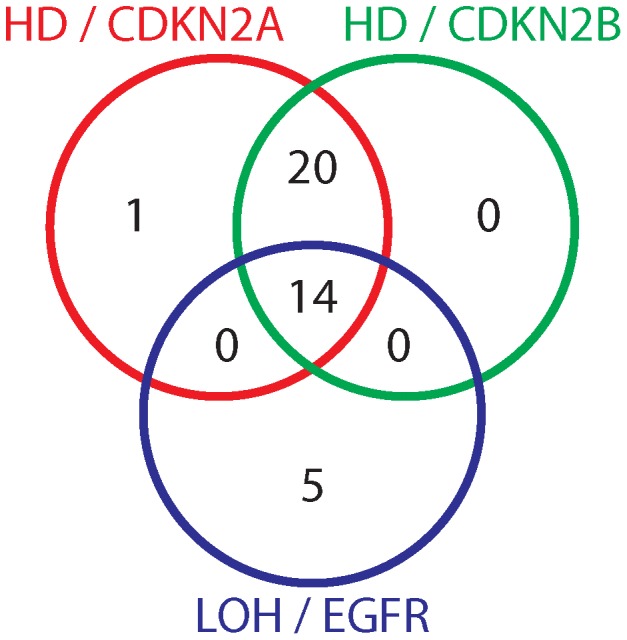
Overlap of genomic events among patients harboring risk allele at rs1015793. Venn diagram of patients that were homozygous for the risk allele at rs1015793, and displayed aberrations at EGFR and CDKN2A/B.

Among the UMU samples, 60 samples were homozygous for the risk allele at the EGFR variant rs1015793 (which was used as a surrogate for the risk variant rs17172430). Of these, 19 displayed LOH at the EGFR locus, and 35 displayed HD at the CDKN2A locus. Fourteen of the 60 displayed both HD at CDKN2A/B and LOH at the EGFR locus (Fig. 5).

The results are summarized in Table 5.

## Discussion

Molecular profiling of glioma has identified several important pathways that characterize the different histopathological types of glioma. Increased insight into glioma biology is important to help understand its etiology and initiation processes, which in turn may enable development of preventive and therapeutic strategies. In the present study, we have identified correlations between germline EGFR gene variants (rs17172430 and rs11979158) and loss of heterozygosity (LOH) at the EGFR locus as well as homozygous deletion at the CDKN2A/B locus. This is indicative of a functional effect of a germline variant on tumor progression. Discoveries in genetic etiology have been important for the development of novel treatments in other cancers, such as PARP-2 inhibitors in breast cancer patients carrying mutations in the BRCA1 gene [17].

Early studies showed two major pathways of glioma progression, characterized by EGFR amplification [18] and TP53 alterations [19], respectively. The two pathways were anticipated to be mutually exclusive. More recently, additional genetic signatures have been discovered, such as co-deletion of chromosomal arms 1p and 19q in oligodendroglioma [20] and IDH1 mutations, where the latter is typical among low grade tumors [21]. The cancer genome atlas research network (TCGA) has successfully characterized 206 glioblastoma cases by comprehensive analysis of DNA copy number, gene expression and DNA methylation aberrations [6]. They identified three major pathways that are central to glioma progression. As EGFR and CDKN2A/B each is an early actor in at least one of these three pathways, the results of our study relate to all three of the TCGA presented pathways.

We have investigated 13 germline gene variants, annotating 8 genes associated with glioma susceptibility (Table 1). Variants of the CCDC26 and PHLDB1 genes have predominantly been associated with low grade glioma, and there is a clear correlation between these variants and IDH1 mutation status [1]. Variants of the TERT and RTEL genes are predominantly associated with glioblastoma [12], [13], whereas variants of the CDKN2A and EGFR genes are associated with overall glioma risk, not with a specific subtype. The UMU dataset used in the discovery phase in our study included various histological subtypes of glioma – hence genetic aberrations found associated with germline variants in this dataset are likely to be aberrations common between glioma subtypes. Aberrations common between glioma subtypes are most likely early events, and therefore of general importance to glioma etiology.

In this study, we investigated two variants annotating the CDKN2A/B genes (rs1412829 and rs4977756). We found no significant associations between these variants and somatic copy number alterations anywhere in the genome. This is similar to the findings of another recent study, where the same variants were investigated in relation to copy number alterations of the CDKN2A/B genes, and no associations were found [22].

**Figure 6:**
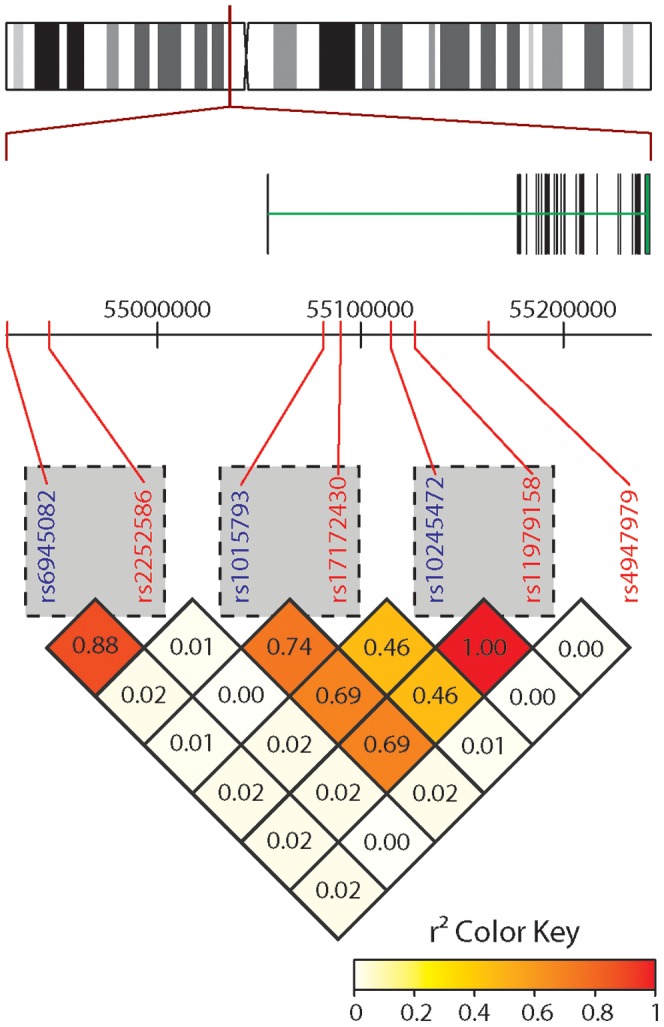
EGFR gene structure. Schematic diagram of the EGFR gene structure, marking all gene variants included in the study and their internal LD structure. Pairs of risk variants (red) and surrogate markers used in the UMU data (blue) are marked with dashed rectangles.

We report a correlation between a risk variant in EGFR intron 1 (rs17172430) and LOH at the EGFR locus. This is in contrast to the report by Sanson et al [1], in which they found no significant correlation between EGFR risk variants and EGFR amplification, determined by fluorescence in situ hybridization (FISH). The discrepancy between these findings may in part be explained by the different methods used. Our approach allowed for investigation of allele-specific loss and gain, whereas FISH is not designed to detect allele-specific events (such as copy number neutral LOH). Moreover, the two studies have investigated overlapping but different sets of gene variants. The variants are all in disequilibrium with each other, to a certain degree (Fig. 6), but it is not surprising that one variant correlates to a specific genomic event whereas the others do not.

Figure 3 reveals almost identical frequencies of the two events LOH and ‘simultaneous LOH and increased copy number’ at the EGFR locus (frequency peaks close to the middle of chromosome 7); 26% and 25% respectively. With this in mind, and considering that the EGFR risk variant rs17172430 is associated with an increased frequency of LOH at the EGFR locus, one would expect it to be associated also with an increased frequency of ‘simultaneous LOH and increased copy’ number at the same locus. The fact is, we observe a border line significant association (P = 0.051) between the two. The discrepancy between the frequencies of LOH and ‘simultaneous LOH and increased copy number’ at the EGFR locus in the UMU dataset is made up by only one patient, who is classified as positive for LOH but negative for ‘simultaneous LOH and increased copy number’ at the EGFR locus.

EGFR is a large gene (188 kb) with a complex haplotype structure. Intron 1 alone, harboring both rs17172430 and rs11979158, consists of >122 000 bases. Our findings imply that variation in this region has a functional role. However, this needs to be investigated further, for example by targeted re-sequencing of the region, to explore the possibility of germline functional mutations in linkage disequilibrium with the identified risk genotype.

The other main findings of our study were correlations between the EGFR risk genotypes (rs17172430 and rs11979158) and homozygous deletions of CDKN2A/B. Genetic events in CDKN2A/B and EGFR often co-occur, thus it is difficult to disentangle whether these are independent events, or whether the CDKN2A/B events are secondary to the EGFR event. Table 5 lists three separate associations between rs17172430 and HD at 9p21 (i.e. the CDKN2A/B locus); these should not be considered independent. The separate listings reflect both that two different approaches were used to analyze the data, and the fact that the global approach often picked up adjacent regions that were separated due to frequency drops caused by genomic breakpoints in just a few patients (Table S1). Figure 4 provides a clear illustration of the association between rs1015793 (used as a surrogate for rs17172430 in UMU data) and HD at 9p21 locus.

Several associations from the discovery phase were not significant in the validation set. The UMU sample set (used for discovery) contained all different subsets of glioma, whereas the TCGA sample set (used for validation) contained only glioblastoma. Because of this, the validation procedure would have discarded any associations between germline gene variants and somatic copy number aberrations that were mainly evident among other lower graded glioma. However, as there appears to be no such events found in the discovery phase (Table S1 and S2), we do not believe this in an issue in this case.

Estimating genome-wide copy numbers from SNP-chip genotype data from tumor samples is complicated by the facts that tumor cells may not be diploid and that samples many times contain DNA from both tumor and stromal cells. The ASCAT algorithm solves both these inherent difficulties and allows for genome-wide allele-specific analysis of copy number from tumor samples [15]. As LOH events are important in cancer development, allele-specific copy number data can be very important. In this study, we used two approaches to explore correlations between germline gene variants and somatic aberrations; one global and one focused on selected genes of interest. The global approach was exploring the possibility that the germline gene variants could be associated to somatic events anywhere in the genome. This approach did not provide evidence for a higher frequency of somatic aberrations in patients with a specific germline variant. However, this analysis has clear power limitations and was thus complemented by a focused genes-of-interest analysis.

In conclusion, we have found correlations between EGFR gene variants and somatic aberrations of both EGFR and CDKN2A/B. We believe these variants may have a driving effect on glioma progression, and thus provide a novel lead to further understanding of genotype-phenotype correlations in glioma etiology. Additional studies of the direct functional role need to be conducted to elucidate the molecular mechanisms underlying the identified association between germline gene variants and somatic aberrations.

## Supporting Information

Table S1 **Validation results, global approach.** Results from validation of all regions that were selected for validation by the global approach.
(XLSX)

Table S2 **Validation results, genes-of-interest approach.** Results from validation of all genes that were selected for validation by the genes-of-interest approach.
(XLSX)
